# Variant Site-Specific Natural History of Titin-Induced Cardiomyopathy: An International Multicenter Registry

**DOI:** 10.1161/CIRCGEN.125.005422

**Published:** 2026-07-08

**Authors:** Maria Perotto, Cinzia Radesich, Alessia Paldino, Ilaria Gandin, Job A.J. Verdonschot, Yuri Kim, Iswaree Devi Balakrishnan, Sophie L.V.M. Stroekes, Kristen A. Medo, Marta Gigli, Stefania Lenarduzzi, Giorgia Girotto, Brian L. Claggett, Enrico Ammirati, Andrea Barison, Edoardo Bertero, Elena Biagini, Marco Canepa, Stefano Carugo, Sharlene M. Day, Raffaello Ditaranto, Andrea Faggiano, Alessandra Fornaro, Cinzia Forleo, Piero Gentile, Andrea Igoren Guaricci, Adam S. Helms, Carolyn Y. Ho, Maria Iascone, Annamaria Iorio, Sadiya S. Khan, Kimberly Y. Lin, Luisa Mestroni, Iacopo Olivotto, Anjali T. Owens, Flavio L. Ribichini, Joseph W. Rossano, Supriya Shore, Martina Setti, Matthew T. Wheeler, Matthew R.G. Taylor, Lisa D. Wilsbacher, Upasana (Paz) Tayal, Victoria N. Parikh, Stephane Heymans, Sanjay K. Prasad, Italian TTN Consortium and DCM/AC SHaRe Consortium, Gianfranco Sinagra, Marco Merlo, Neal K. Lakdawala, Matteo Dal Ferro

**Affiliations:** 1 Cardiovascular Department, Azienda Sanitaria Universitaria Giuliano Isontina (ASUGI), University of Trieste, Italy (M.P., C.R., A.P., M.G., G.S., M.M., M.D.F.).; 2 Member of the European Reference Network for Rare, Low-Prevalence, or Complex Diseases of the Heart (ERN GUARD-Heart) (M.P., C.R., A.P., J.A.J.V., M.G., E. B., R.D., S.H., G.S., M.M., M.D.F.).; 3 Biostatistics Unit, University of Trieste, Italy (I.G.).; 4 Department of Cardiology, Cardiovascular Research Institute (CARIM), Maastricht University Medical Centre, the Netherlands (J.A.J.V., S.L.V.M.S., S.H.).; 5 Department of Medicine, Brigham and Women’s Hospital, Harvard Medical School, Boston, MA (Y.K., I.D.B., B.L.C., C.Y.H., N.K.Y.L.).; 6 Department of Cardiology, National Heart Centre Singapore (I.D.B.).; 7 Cardiovascular Institute & Adult Medical Genetics Program, University of Colorado Anschutz Medical Campus, Aurora (K.A.M., L.M., M.R.G.T.).; 8 Institute for Maternal & Child Health-IRCCS (S.L., G.G.).; 9 Dept of Medicine, Surgery and Health Sciences, University of Trieste, Italy (G.G., G.S., M.M.).; 10 De Gasperis Cardio Center & Transplant Center, Niguarda Hospital, ASST Grande Ospedale Metropolitano Niguarda, Milan, Italy (E.A., P.G.).; 11 Cardiology Division, Fondazione Toscana Gabriele Monasterio, Pisa, Italy (A.B.).; 12 Cardiovascular Unit, Department of Internal Medicine, University of Genova, Italy (E. B., M.C.).; 13 Cardiology Unit, IRCSS Azienda Ospedaliero-Universitaria di Bologna, Italy (E. B., R.D.).; 14 Department of Cardio-Thoracic-Vascular Diseases, Foundation IRCCS Ca’ Granda Ospedale Maggiore Policlinico (S.C., A. F.).; 15 Department of Clinical Sciences & Community Health, University of Milan, Italy (S.C.).; 16 Division of Cardiovascular Medicine, Perelman School of Medicine, University of Pennsylvania, Philadelphia (S.M.D., A.T.O.).; 17 Cardiomyopathy Unit, Careggi University Hospital, Florence, Italy (A. F., I.O.).; 18 Interdisciplinary Department of Medicine (DIM), School of Medicine, University of Bari Aldo Moro, Italy (C.F., A.I.G.).; 19 Cardiology Department, Papa Giovanni XXIII Hospital, Bergamo, Italy (M.I., A.I.).; 20 Department of Internal Medicine, University of Michigan, Ann Arbor (A.S.H., S. S.).; 21 Department of Medicine-Cardiology, Feinberg School of Medicine, Northwestern University, Chicago, IL (S.S.K., L.D.W.).; 22 Department of Pediatrics, The Cardiac Centre, The Children’s Hospital of Philadelphia, Perelman School of Medicine at the University of Pennsylvania, Philadelphia (K.Y.L., J.W.R.).; 23 Division of Cardiology, Department of Medicine, University of Verona, Italy (F.L.R., M.S.).; 24 Stanford Center for Inherited Cardiovascular Disease, CA (M.T.W., V.N.P.).; 25 Royal Brompton & Harefield Hospitals, Guy’s and St. Thomas’ NHS Foundation Trust, London, United Kingdom (U.T., S.K.P.).; 26 National Heart Lung Institute, Imperial College London, United Kingdom (U.T.).

**Keywords:** arrhythmias, cardiac, cardiomyopathy, dilated, genotype, heart failure, prognosis, Titin-bands

## Abstract

**BACKGROUND:**

Titin truncating variants (*TTN*tv) represent the most common genotype underlying dilated cardiomyopathy but are also detected in the general population, exhibiting incomplete penetrance and marked phenotypic variability. This heterogeneity complicates clinical interpretation and risk stratification. Emerging molecular evidence suggests that truncating location within the gene may influence disease mechanisms. We aimed to investigate whether *TTNt*v location also affects clinical phenotype and prognosis.

**METHODS:**

We established an international multicenter registry of phenotypically affected carriers of pathogenic or likely pathogenic *TTN*tv. Patients were classified into 3 groups: A-band, Z/I-band, and M-band. A case-control study assessed the enrichment of *TTN*tv across regions. The primary outcome was a composite of all-cause mortality and heart transplantation. Secondary outcomes included: (1) sudden cardiac death or major ventricular arrhythmias and (2) heart failure-related death/heart transplantation/left ventricular assist device implantation.

**RESULTS:**

The study included 467 patients (81% probands, 73% men, median age 47 years, 81% dilated cardiomyopathy phenotype). Most carried *TTN*tv in the A-band (80% versus 15% Z/I-band and 5% M-band). All groups showed enrichment compared with GnomAD, with greater Bayesian-estimated penetrance for A-band variants. Over a median follow-up of 83 months, the primary end point was similar across groups. However, the risk of sudden cardiac death/major ventricular arrhythmias was significantly higher in M-band carriers (45% M-band versus 23% Z/I-band versus 12% A-band; *P*=0.001), especially as the first disease manifestation. Band location independently predicted sudden cardiac death/major ventricular arrhythmias risk, whereas left ventricular ejection fraction was predictive only in A- and Z/I-band groups.

**CONCLUSIONS:**

*TTN*tv are differently enriched across the gene in patients with dilated cardiomyopathy/non-dilated left ventricular cardiomyopathy. Penetrance and risk of sudden cardiac death/major ventricular arrhythmias differ according to variant location, supporting the *TTN* truncation site as a parameter that should be considered for personalized risk stratification in *TTN* cardiomyopathy.

Cardiomyopathy (CMP) caused by heterozygous titin truncating variants (*TTN*tv) in the *TTN* gene represents the most prevalent form of inherited CMP.^1^ This underscores the critical importance of deeply understanding its natural history and prognosis.^2^ However, despite significant progress, several key aspects of the clinical significance of *TTN*tv remain poorly characterized.

*TTN*tv are found in the general population in up to 0.5% to 1% of cases.^1,3,4^ It is known that only those affecting constitutively expressed exons (percentage spliced in [PSI] >90%) of cardiac isoforms cause CMPs,^3–5^ but the impact of the location of pathogenic variants across the *TTN* gene on clinical outcomes has been only marginally explored previously. Indeed, *TTN*tv in different exons show different disease association and estimated penetrance in case-control studies, with variation according to the corresponding protein domain (Z-disk, I-band, A-band, M-band), and less evidence exists regarding *TTN*tv located outside the A-band.^4,6,7^

A comprehensive understanding of the pathogenicity of all *TTN*tv,—not just those affecting the A-band—is of utmost importance to guide clinical decision-making. To this end, it should be considered that available genetic and clinical evidence on *TTN*tv-related CMP (*TTN*-CMP) may suffer from several biases. First, previous case-control studies of disease association were conducted almost exclusively in dilated cardiomyopathy (DCM) probands, in some cases excluding individuals with cardiac devices.^8^ These studies produced conflicting results: while generally agreeing on the pathogenicity of A-band *TTN*tv and their association with DCM, they obtained inconsistent data for variants located outside the A-band,^4,6^ showing no or weaker disease association. This uncertain role of *TTN*tv outside the A-band led to a second bias: non–A-band *TTN*tv may be interpreted inconsistently across different genetic laboratories, potentially going unreported, thus undermining a correct estimate of their true prevalence. In addition, the *TTN* gene was not historically included in multigene panels for arrhythmic or non-dilated CMPs.^9^ Currently, common clinical practice tends to consider and therefore look for *TTN*tv only in cases of DCM, excluding other forms of left-sided non-hypertrophic CMPs or suspected channelopathies. As a result, the genetic and phenotypic association of *TTN*tv, and especially non–A-band *TTN*tv, across the full spectrum of left-sided non-hypertrophic CMPs remains incompletely characterized.

To address these limitations, our large international study on *TTN*-CMP enrolled patients with any form of non-hypertrophic CMP, thereby extending the scope of analysis beyond DCM. First, variant-disease association and estimated penetrance (Bayesian estimation^10,11^) of all *TTN*tv in high-PSI exons were evaluated, according to truncation site, in a case-control study using a newly built case cohort, not restricted to DCM-only probands, and with an ascertainable arrhythmic burden (∼40% Implantable Cardioverter Defibrillators -ICD- recipients). Then, clinical features and natural history of the disease were described and stratified by the location of the likely pathogenic/pathogenic variant within the *TTN* gene. We focused on arrhythmic risk, as specific predictive factors for major ventricular arrhythmias (MVA)/sudden cardiac death (SCD) in *TTN*-CMP have yet to be adequately addressed.^12,13^

## Methods

### Study Population: International Band-Specific *TTN*tv Registry (Italian TTN Consortium and Sarcomeric Human Cardiomyopathy Registry [SHaRe] ACM/DCM)

The study enrolled patients from 16 international CMP centers and included patients aged ≥16 years, carrying a heterozygous *TTN*tv and affected by any type of non-hypertrophic CMP. Phenotype-negative carriers were excluded. Only carriers of *TTN*tv located in exons with >90% PSI and predicted to be likely pathogenic/pathogenic according to the American College of Medical Genetics and Genomics guidelines^14^ were collected by each participating center (Supplemental Methods), and anonymized to create the international registry. The study complies with the Declaration of Helsinki and was approved by the Ethics Committee (institutional review board) of Trieste University Hospital (43_2009 Em. 02 dd. 22/06/2022) and by the institutional review board/Ethics Committee of each participating center. For SHaRE, data harmonization was performed by Boston Advanced Analytics using its Aurora software, storing data in Microsoft SQL Server.

Because of the sensitive nature of the data collected for this study and restrictions imposed by ethics approvals, the data sets are not publicly available. Requests to access the data from qualified researchers trained in human subject confidentiality protocols may be directed to the corresponding author, subject to appropriate approvals.

### Genetic Testing and Band Description

Genetic testing was performed at participating centers or at accredited genetic laboratories by next generation sequencing following site-specific protocols, with different CMP-validated multigene panels including *TTN*. Genetic data were collected for probands and available family members, and any additional variant detected in other CMP-related genes was reported. Individuals with a *TTN*tv and a likely pathogenic/pathogenic variant in another CMP gene were excluded. All rare (minor allele frequency below 0.000084 in gnomAD v4.1^15^) *TTN*tv (nonsense, frameshift, and essential splice site variants) were reanalyzed and localized into their respective band in the canonical transcript (NM_001267550.2):^3,16^ the Z-disk region (exons 1–10; 12–28), I-band (constitutive exons 29–44, 47–49, 219–252), A-band (252–355), A to M junction (exons 356–357), or M-band (exons 358–363). Then, they were further grouped into: (1) N-terminal group, comprising Z-disk and I-band exons (hereafter called Z/I-band); (2) A-band; and (3) C-terminal group, including the A to M junction and M-band exons (hereafter called M-band).

### Enrichment Analysis and Truncating Variant Frequencies

Enrichment testing was performed on probands alone^8^ using a study case cohort of 2000 patients with DCM/nondilated left ventricular (LV) cardiomyopathy (NDLVC), as further detailed in the Supplemental Methods. The burden of rare (gnomAD minor allele frequency <0.000084) *TTN*tv in exons with PSI>90% in the study case cohort was calculated, and compared with a control cohort composed of individuals from the gnomAD, v4.1 population (N=798 504). Allele frequency (number of distinct *TTN*tv normalized by coding length in base pairs) and carrier frequency (number of *TTN*tv carriers normalized by coding length in base pairs) were also calculated in cases and controls, and compared.^10,11^ The comparison was made separately for different *TTN* bands and their longer exons (exon 48 for the I-band, the so-called N2B unique sequence [N2Bus], 326 for the A-band, and 358 for the M-band); see Supplemental Methods.

### Phenotypic Characterization

Each participating center collected demographic, family history, clinical history, and treatment data for each patient at baseline (defined as the first medical evaluation performed upon disease presentation) and at the last available evaluation before June 2024. A detailed list of collected data and methods is presented in the Supplemental Methods.^2,3,8,17,18^

### Study Outcomes

The primary outcome of the study was a composite of all-cause mortality (D) and heart transplantation (HT). Specific secondary outcomes were the following:

1. Arrhythmic outcome: SCD/first MVA, including baseline events.
2. Heart failure (HF) outcome: HF-related death/HT/LV assist device implantation.

### Statistical Analysis

For the analyses, follow-up ended at the date of the first outcome or the latest available contact with the patient. For the SCD/MVA outcome, cumulative incidence curves were obtained, accounting for death from other causes and HF/HT as competing events and compared using the Gray test. The same approach was applied to the HF/HT end point, where death from other causes and SCD/MVA were considered competing events. Cause-specific Cox proportional hazards mixed-effects models were also obtained to include other risk factors. To account for familial relationships, family membership was treated as a random effect. Patient origin, categorized as Italian or non-Italian, was addressed using a binary stratification term. To better identify predictive factors for SCD/MVA risk in patients with *TTN*-CMP, we conducted several multivariable analyses on the subgroup of patients who experienced the events and who had available data for all covariates. Detailed statistical methods are reported in the Supplemental Methods.

## Results

### Genetic Description of the Study Population and Genetic Analysis

A total of 467 patients with *TTN*tv were enrolled in the study, of which 125 cases belonged to 48 families, each with 2 to 5 individuals, while the remaining patients were unrelated. Collectively, these patients carried 304 distinct *TTN*tv (Table S1). *TTN*tv in the A-band were present in 375 (80%) cases, in the Z/I-band in 69 (15%) cases, and in the M-band in 23 (5%) cases. The distribution of variants across *TTN* gene is reported in Figure 1. The most frequently involved exons were exon 48 (n=25 patients) in the Z/I-band (encoding N2Bus), exon 326 (n=110) in the A-band, and exon 358 (n=18) in the M-band—the 3 largest exons of titin.

**Figure 1.**
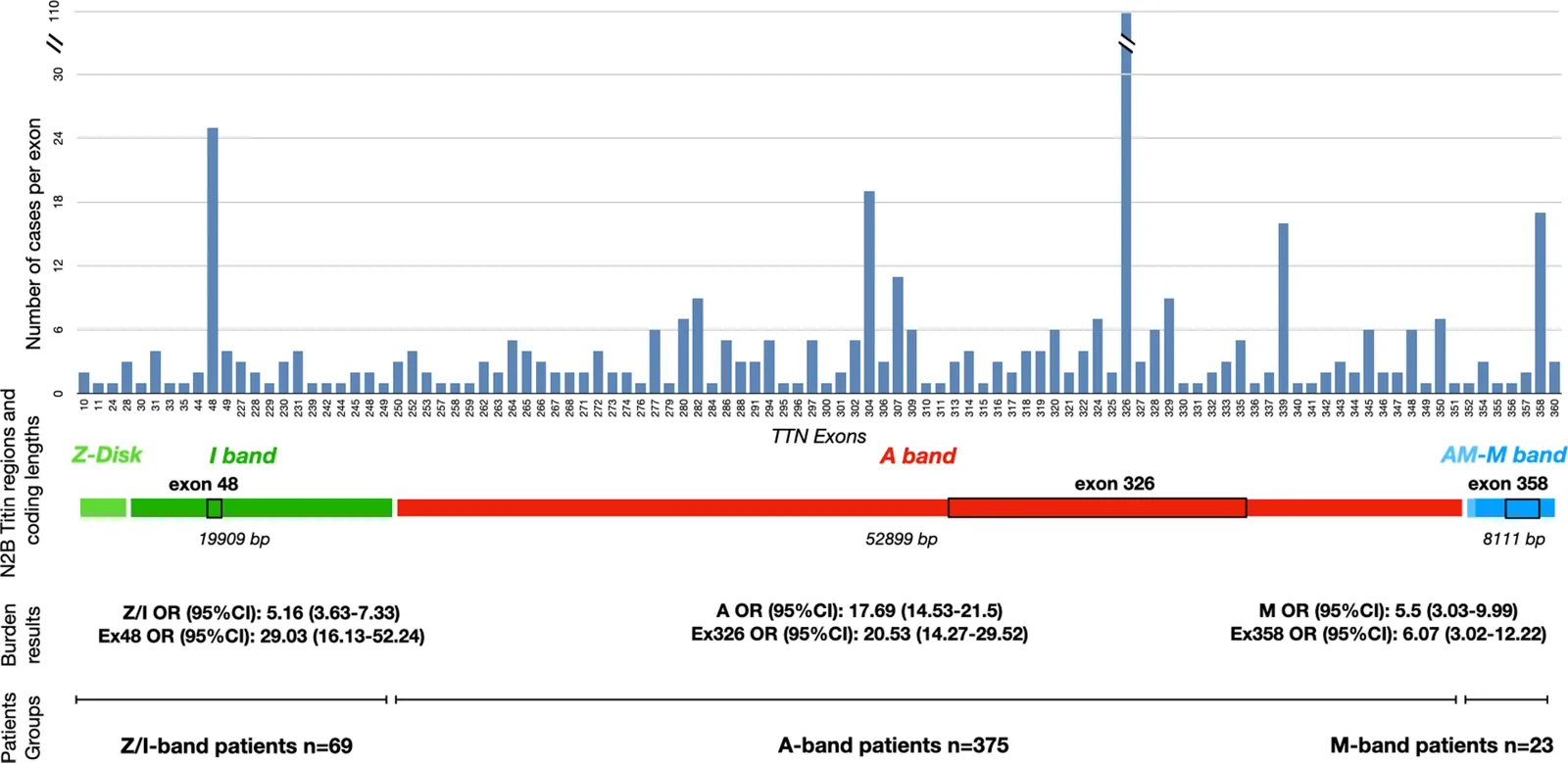
**Distribution of titin truncating variants (*TTN*tv) in the study population across different regions and exons of titin gene. Top**, Histogram showing *TTN* exons and number of cases carrying TTNtv in each exon. **Middle**, Picture of different bands of titin protein (cardiac main isoform, N2B) with most prevalent exons. Coding length in base pair is also reported. **Bottom**, Patients grouping according to different protein regions with burden testing results. Ex indicates exon.

Band- and exon-specific burden testing of *TTN*tv in our DCM/NDLVC case cohort compared with the gnomAD database v4.1 confirmed the enrichment of *TTN*tv in all bands. In absolute terms, the A-band and 2 single exons (48 and 326) exhibited the highest odds ratio and etiologic fraction for disease (Figure 1 and Table S2). When evaluating the allele frequency and carrier frequency in each population (cases and controls), the gnomAD population showed lower frequencies of rare alleles and carriers in the A-band, exon 48, and exon 326, compared with other bands and exons. Conversely, the DCM/NDLVC cases exhibited the highest frequencies of rare alleles and carriers in the A-band, exon 48, and exon 326 (Table S3). This symmetrical pattern confirms a higher estimated penetrance of *TTN*tv located in these regions.^10,11^

### Baseline Characteristics

Table 1 shows the baseline characteristics of the overall population of 467 patients with *TTN*tv, grouped by variant location. Our population predominantly consisted of patients of European ancestry (91%), probands (81%), and men (73%). The median age at enrollment was 48 (37–57) years for probands and 43 (41–55) years for family members (*P*=0.10). Among probands, those carrying Z/I-band variants were older at diagnosis (median age, 54 years) compared with those with A-band variants (median age, 45 years; *P*=0.004). The prevalence of comorbidities that can contribute to LV systolic dysfunction (hypertension, diabetes and nonobstructive coronary artery disease) was low (<20%) across the entire cohort, and similar in the 3 band groups (*P*=0.6). The most common pattern of remodeling at presentation was DCM (81%); 19% of patients presented with NDLVC.

**Table 1.**
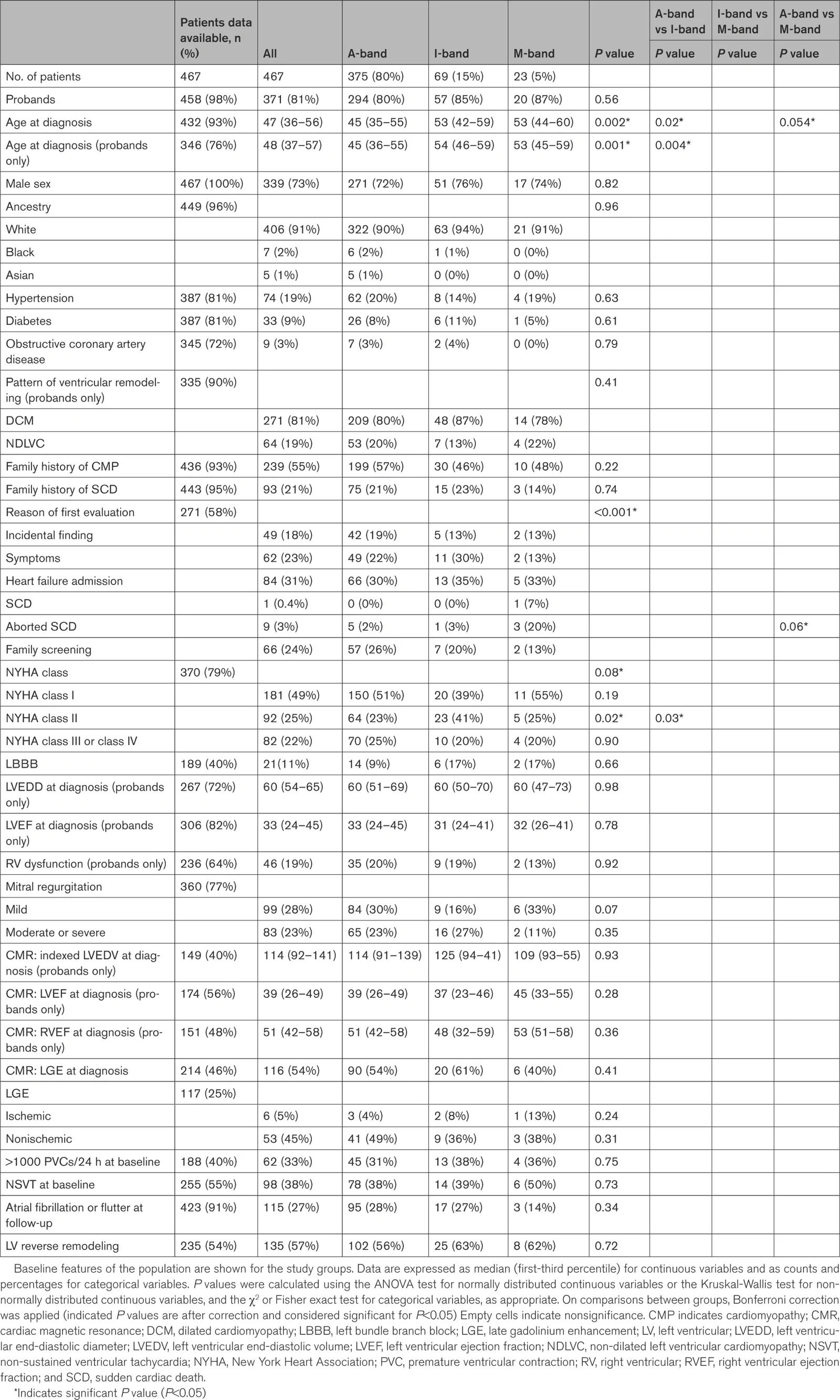
Main Baseline and Follow-Up Characteristics of the Study Population

Overall, the most frequent reason for the first medical evaluation was HF admission (31%) for all patients. However, aborted SCD tended to be a more common presentation in patients with M-band *TTN*tv compared with the other 2 groups (n=3 [20%] in the M-band versus n=5 [2%] in the A-band and n=1 [3%] in the Z/I-band; *P*=0.06 for M-band versus A-band).

The first evaluation for family screening was twice as likely in the A-band compared with the M-band. Median LV dimensions (LV end-diastolic diameter, 60 mm [54–65]) and function (LV ejection fraction [LVEF], 34% [25–45]) at baseline did not differ significantly among groups.

### Follow-Up and Outcomes

In the entire cohort, follow-up data were available for 436 (93%) patients. Fifty-seven percent of patients achieved LV reverse remodeling under medical therapy over 6 months to 1 year, with similar percentages across different groups. During a median follow-up of 83 months (interquartile range, 31–155), 65 (15%) patients died (n=46, 4 of whom died after HT) or underwent HT (n=23), 84 (19%) experienced SCD/MVA, and 62 (14%) HF events (Table 2 and Table S5). The frequency of the primary outcome was similar between patients with truncating variants in different bands of titin (Figure S1).

**Table 2.**
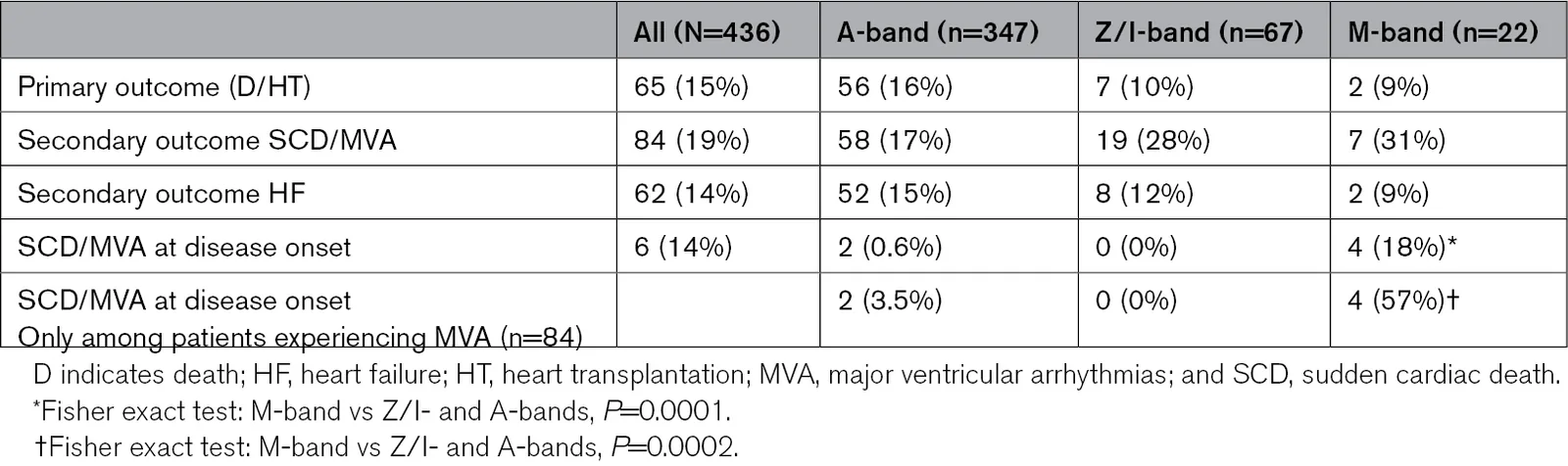
Clinical Outcomes in Patients With *TTN*tv Based on the Variant Location

Overall, SCD/MVA events were more frequent in patients with *TTN*tv corresponding to the Z/I- and M-bands compared with the A-band. Comparison of cumulative incidence curves for arrhythmic outcomes over 10 years revealed a significantly higher risk of events in patients with M-band *TTN*tv (*P*<0.001; cumulative incidence estimate, 45%; 95% CI, 12%–75%) compared with the Z/I-band (23%; 95% CI, 12%–37%) and A-band (12%; 95% CI, 7.9%–16%) (Figure 2).

**Figure 2.**
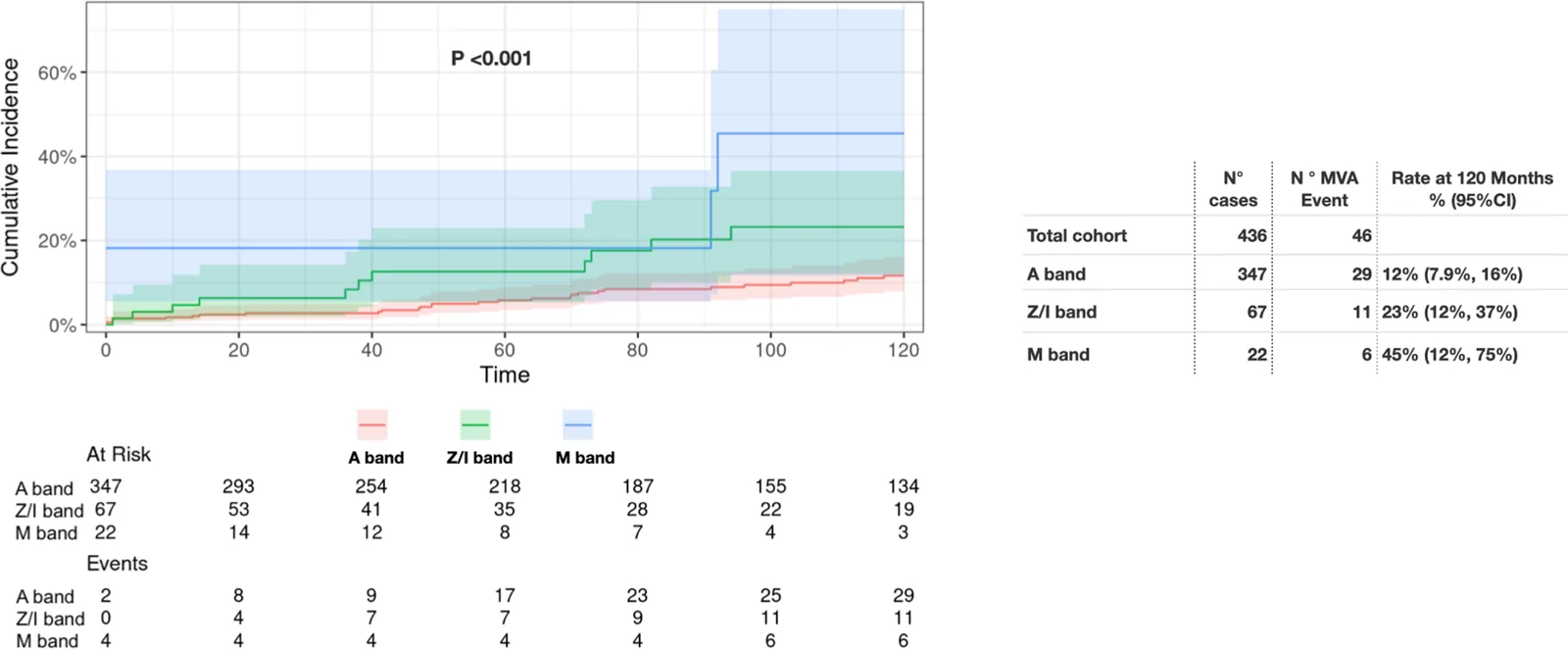
**Cumulative incidence of major ventricular arrhythmia and sudden cardiac death stratified by titin truncating variants (*TTN*tv) location.** Cumulative incidence function curves depict the 10-year risk of major ventricular arrhythmia (MVA)/sudden cardiac death according to *TTN*tv location across A-band, Z/I-band, and M-band groups. A statistically significant difference in event rates between groups was observed (*P*<0.001), with the highest risk in the M-band, followed by the Z/I-band and A-band.

Patients with M-band *TTN*tv were characterized by a higher incidence of SCD/MVA events at clinical presentation (18% versus 0.5% in the Z/I- and A-bands; *P*=0.0001). Indeed, SCD/MVA at the time of diagnosis accounted for over half (57%) of the total SCD/MVA events in patients with M-band *TTN*tv (Table 3). Even after excluding patients who experienced SCD/MVA at disease onset from the analysis, a significant difference in SCD/MVA risk was still observed across the 3 band groups, even when considering probands only. However, the low number of events limited drawing band-specific conclusions (*P*=0.03 and *P*=0.02; Figures S2 and S3). Secondary prevention Implantable Cardioverter Defibrillators were implanted more frequently in patients with *TTN*tv corresponding to the M-band (23% versus 4% in the A-band and 6% in the Z/I-band; *P*=0.003, Table S4). However, primary prevention Implantable Cardioverter Defibrillator implantation did not vary between patients based on the location of the *TTN*tv.

**Table 3.**
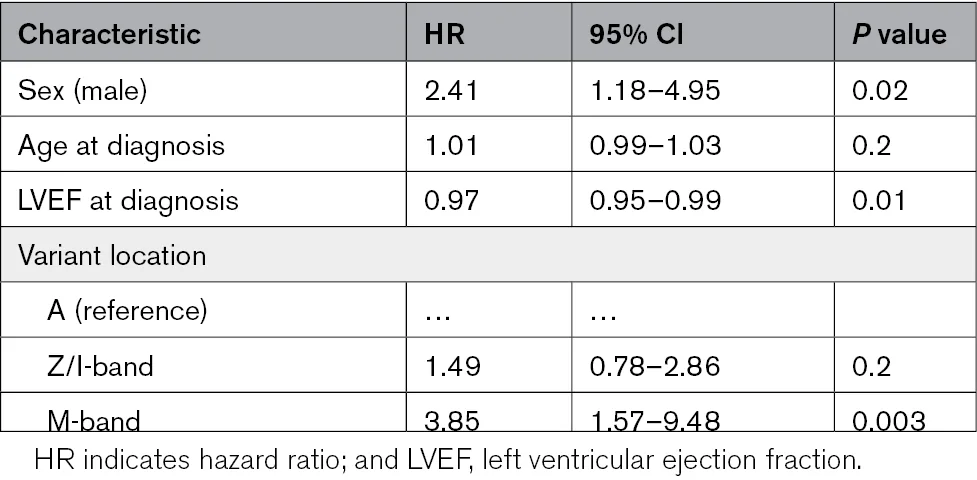
Cox Regression Model for Ventricular Arrhythmias in Patients With *TTN*tv

Cumulative incidence function curves showed higher rates of HF events in the A- and Z/I-band groups compared with the M-band group (with no HF events observed). However, this difference was not statistically significant (*P*=0.5; Figure S4).

### Multivariable Analyses for Arrhythmic Risk Prediction

In a univariate analysis, the presence of an M-band *TTN*tv compared with an A-band *TTN*tv was associated with a higher SCD/MVA risk (hazard ratio [HR], 4.1 [95% CI, 1.6–10.6]; *P*=0.003). This association remained significant in a multivariable model after adjusting for sex, age at diagnosis, and LVEF at first evaluation (HR, 3.85 [95% CI, 1.57–9.48]; *P*=0.003; Table 3), and also after further adjusting for diabetes, hypertension, and coronary artery disease (Figure 3). There was no statistical difference in SCD/MVA risk when comparing the Z/I-band with the A-band.

**Figure 3.**
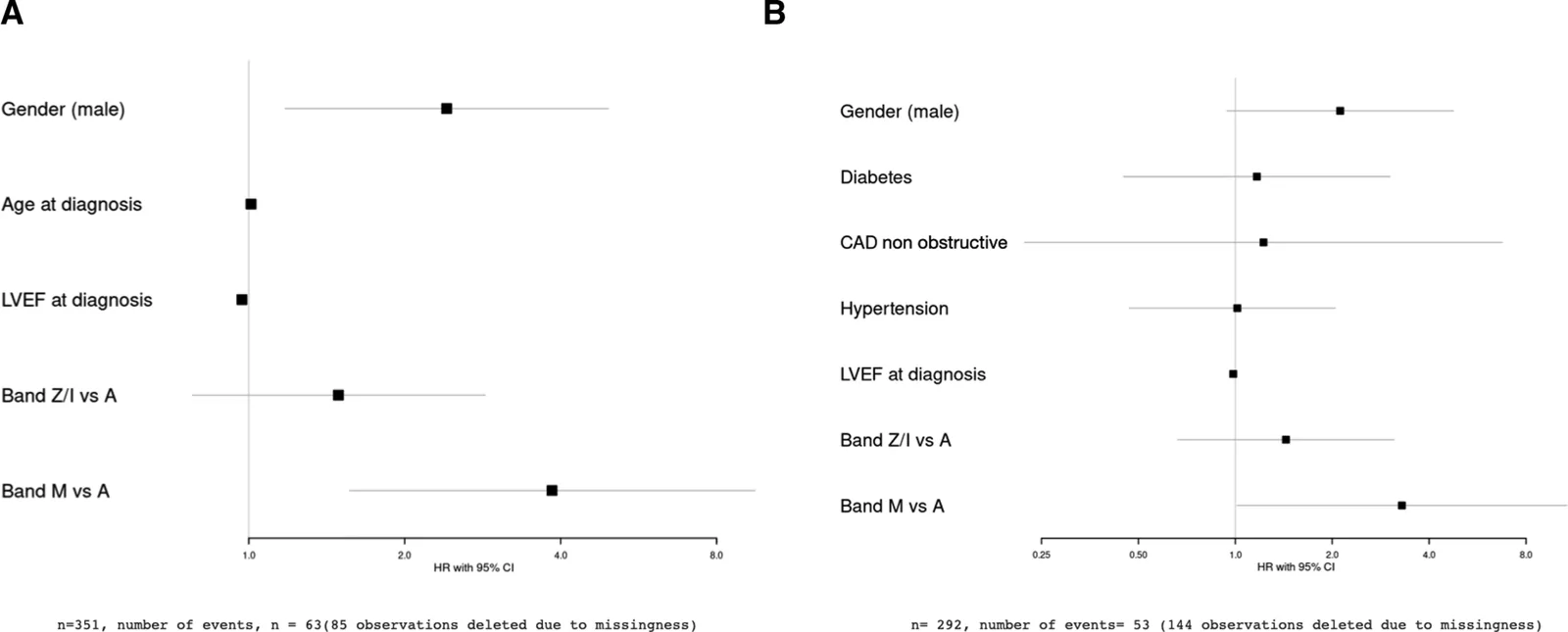
**Multivariable predictors of major ventricular arrhythmia and sudden cardiac death.** Forest plots showing hazard ratios (HRs) with 95% CIs from multivariable Cox regression analyses for the composite outcome of major ventricular arrhythmia/sudden cardiac death. **A**, Includes sex, age at diagnosis, left ventricular ejection fraction (LVEF) at diagnosis, and titin truncating variants (*TTN*tv) location (Z/I-band vs A-band; M-band vs A-band). **B**, Presents an expanded model including sex, diabetes, non-obstructive coronary artery disease (CAD), hypertension, LVEF at diagnosis, and *TTN*tv location.

At the multivariable analysis, LVEF was associated with SCD/MVA risk (HR, 0.97 [95% CI, 0.95–0.99]; *P*=0.011; Table 3) in the entire cohort. The role of LVEF at first evaluation was investigated separately in the 3 groups (A-band, Z/I-band, and M-band). LVEF at first evaluation was associated with the risk of SCD/MVA in individuals carrying A-band (HR, 0.97 [95% CI, 0.94–1]; *P*=0.02) and Z/I-band *TTN*tv (HR, 0.94 [95% CI, 0.94–1]; *P*=0.05). In these 2 groups, the effect of LVEF appeared linear, with LVEF <35% as cut-point for risk stratification, especially in the A-band group. Notably, this association between SCD/MVA risk and baseline LVEF was not found in the M-band group (*P*=0.2; Figure S5 and Table S6).

## Discussion

In this band-specific genotype-phenotype correlation study in *TTN*-CMP, we confirmed that *TTN*tv in >90% PSI exons were significantly enriched across all bands of the protein in a distinct case cohort of probands with DCM and NDLVC. In addition, we showed that *TTN*-CMP presents with phenotypic and clinical heterogeneity, not solely as DCM. Finally, we noted that the natural history of the disease was influenced by *TTN*tv location, especially when considering arrhythmic risk. Altogether, these results reflect distinct disease expressivity depending on the site in which the *TTN*tv occurs.

### Bayesian Estimation of Variant-Specific Penetrance of *TTN*tv

From a population genetics perspective, the low allele and carrier frequencies of *TTN*tv in the A-band and N2Bus in gnomAD suggest they are less likely to be tolerated in the general population (negative selection), thus more penetrant and likely leading to CMP when present. Conversely, the relatively higher allele and carrier frequencies of *TTN*tv affecting M-band exons in gnomAD aligns with reduced penetrance for the DCM phenotype compared with A-band *TTN*tv.

In terms of odds ratio values, the results of the band-specific burden analyses align with those from previous studies for the A-band, the N2Bus, and exon 326, showing the strongest association also in this case cohort.^4–7^ However, differences in relative prevalence emerge: in our DCM/NDLVC case cohort (composed of probands with only mild median LV dilatation), M-band *TTN*tv showed twice the prevalence found in previous studies (0.55% versus 0.24% in Schafer et al^4^ and 0.25% in Vatta et al^7^), whereas A-band *TTN*tv were significantly less prevalent than in Schafer et al^4^ and Mazzarotto et al.^8^ As this observation is based on a low number of cases, the observed 2-fold higher burden of patients with M-band *TTN*tv in this case cohort warrants further validation, before being considered a direct association of M-band TTNtv with less dilated, more arrhythmic phenotypes.

Collectively, *TTN*tv located in the M-band may recognize a more complex etiology, characterized by lower and more heterogeneous penetrance, despite high-PSI exons, likely reflecting a greater contribution of both non-genetic external modifiers, or distinct (poly)genic modifiers. This complexity makes their clinical risk stratification and prognostic assessment less deterministic and more variable at the single-patient level.

### Band-Related Clinical Profiles and Risks

#### M-Band

Patients with M-band *TTN*-CMP are rarely encountered in clinical practice. In this study, they showed no differences in baseline presentation compared with patients with *TTN*tv in other bands, with only a trend toward more preserved LV dimensions and function, and more common New York Heart Association class I presentation. The main finding of this study is that unrelated probands carrying *TTN*tv in the M-band showed a higher rate of SCD/MVA, both as the first manifestation of disease and throughout follow-up. Notably, this risk did not correlate with the degree of LV dysfunction. If confirmed in other studies, this result would be particularly useful for clinicians when stratifying the arrhythmic risk of patients with M-band-related *TTN*-CMP. A proactive arrhythmic risk assessment and therapeutic management in these patients, especially if they are of young age, might be considered even in the presence of only subtle clinical abnormalities.

Previous clinical reports^7,19^ support these findings on the arrhythmogenicity of M-band *TTN* variants. Experimental evidence on how truncated titin peptides integrate in a nonhomogeneous fashion into the sarcomere depending on the site of truncation,^20,21^ and on the kinase activity of the M-band,^22^ provides a molecular hypothesis supporting such clinical differences. Further research is required to clarify whether the observed differences result from variable penetrance of a common pathogenic process (with distinct contribution from putative second hits), or whether M-band *TTN*tv lead to a distinct disease through alternative molecular pathways. Indeed, in contrast to A-band *TTN*tv, which in our cohort showed higher penetrance and more consistent genotype-phenotype correlations, M-band *TTN*tv showed greater interindividual variability in terms of arrhythmic risk. This heterogeneity could also be attributable to a lower variant effect and a stronger dependence on additional modifiers.

Although the presence of undetected second hits (polygenic and environmental) cannot be excluded, the results of this study support the hypothesis that rare M-band *TTN*tv should always be reported in genetic reports, even when detected as secondary findings,^11,23^ to enable comprehensive cardiological screening of probands and their relatives. This could potentially reduce future inclusion bias in clinical registries.

#### A-Band and Z/I-Band

This study strengthens and refines available evidence on A-band *TTN*-CMP, which represents the vast majority of patients diagnosed with DCM. A stronger penetrance of A-band *TTN*tv was confirmed. Interestingly, a robust linear association between SCD/MVA risk and LV function at baseline was found. Baseline LVEF below 35% was identified as an independent predictor of increased risk of SCD/MVA. Previous evidence^13^ also identified this cutoff for a cumulative end point that included SCD/MVA and HF events. The indication for an Implantable Cardioverter Defibrillator for primary prevention in patients with A-band *TTN*tv when LVEF remains below 35% despite optimal medical therapy, as suggested by guidelines, is supported by this study.

Patients with Z/I-band *TTN*tv clinically resemble those with A-band variants, although with later disease onset and higher New York Heart Association class at baseline. *TTN*tv in exon 48 (N2Bus) in the I-band were found to be particularly penetrant. Further studies are needed to determine whether patients with Z-band *TTN*tv exhibit specific phenotypic features.

### Molecular Insights

From a molecular perspective,^16,24^ the A-band provides essential mechanical support to sarcomere function, while both the C-terminal and N-terminal bands contain domains that modulate distinct molecular pathways.^16^ Recent findings from studies on human hearts with DCM caused by *TTN*tv described sarcomeric insufficiency and the presence of truncated titin peptides, which may exert toxic effects. These peptides were more commonly documented for *TTN*tv located outside the A-band^20,21,25^ and may be differentially integrated into sarcomeres. Furthermore, sarcomeric insufficiency is known to be relatively more pronounced in the A-band, especially in exon 326.^20^ Hence, experimental evidence supports the current finding that the clinical expression of *TTN*-CMP may vary depending on the location of the *TTN*tv.

### Limitations

This study has a retrospective design, and the number of patients with M-band *TTN*tv is small despite the international multicenter effort. Although representing the largest cohort to date of sporadic cases with M-band CMP, our results should be interpreted with caution. Some variables were missing in a certain percentage of patients, requiring their exclusion from analyses. In addition, patient recruitment possibly carried clinical and genetic referral bias. Finally, although adjusted for the most common confounders, the analyses of the study were not adjusted for time-varying covariates. Future prospective studies with larger sample sizes are needed to validate these results and refine risk stratification models.

### Conclusions

The natural history of *TTN*-CMP may differ based on the variant site. A-band variants are the most common and highly penetrant, with the risk of SCD/MVA closely linked to severe baseline LV dysfunction, whereas for M-band variants the association between SCD/MVA risk and LV dysfunction is less consistent. This may reflect a distinct genetic penetrance of M-band variants and a more heterogeneous or multifactorial disease substrate. Although the limited number of events in this cohort precludes definitive conclusions, M-band *TTN*tv are associated with an elevated SCD/MVA risk, and all documented SCD/MVA events occurred in the absence of severe LV dysfunction. Pending confirmation in larger, adequately powered cohorts, our data suggest that the location of *TTN*tv should be considered when stratifying arrhythmic risk in patients with *TTN*-CMP. Collectively, these findings highlight the need for personalized diagnostic and therapeutic strategies tailored to band-specific profiles, underscoring the importance of basic research to elucidate distinct molecular mechanisms underlying *TTN*tv-related disease.

## Article Information

### Disclosures

Dr Mestroni is an advisory board member for Tenaya Therapeutics, Rocket Pharmaceuticals, and has received research support from Greenstone Bio, Pfizer, and Alexion. Dr Day reports consulting for Lexicon Pharmaceuticals and Solid Biosciences, participation on a data safety monitoring board for Cytokinetics, and research grants to his institution from Bristol Myers Squibb and Lexicon Pharmaceuticals, unrelated to the present work. Dr Owens reports consulting for Alexion, Avidity, Bayer, Cytokinetics, Corvista, Edgewise, Imbria, Bristol Myers Squibb, Lexeo, Tenaya, and Stealth, and a research grant to his institution from Bristol Myers Squibb. Dr Ammirati is a consultant for Lexeo and ICON and was a consultant for Hotgen Health, Inc. Dr Tayal is a Research Editor at the BMJ. Dr Rossano reports consulting for AskBio, Astellas, Bayer, Bristol Myers Squibb, and Merck. Dr Biagini reports honoraria and advisory board participation for Sanofi-Genzyme, Takeda, Amicus, Chiesi, and BMS. Dr Lakdawala reports consulting fees from Bristol Myers Squibb, Alexion, Cytokinetics, Tenaya, Gemma, Bayer, and Solid. The other authors report no conflicts.

### Supplemental Material

Supplemental Methods

Tables S1–S7

Figures S1–S5

## Supplementary Material
