# Supplementary material for: Variant Site-Specific Natural History of Titin-Induced Cardiomyopathy: An International Multicenter Registry
Source: Circ Genom Precis Med. 2026 Jul 8;19(5):e005422. doi: 10.1161/CIRCGEN.125.005422 (PMC13510828; doi:10.1161/CIRCGEN.125.005422)

Supplemental Material

1. SUPPLEMENTAL METHODS

Participating Centers

The participants of this large international registry were:

1) Italian Centers: University Hospital of Trieste (coordinator center, and member of "Arrhythmogenic CMP and DCM -Sarcomeric Human Cardiomyopathy Registry"- SHaRe AC-DCM registry), Policlinico of Milan, University Hospital of Genova and IRCCS San Martino, Policlinico Sant'Orsola, Fondazione Toscana Gabriele Monasterio, University Hospital Careggi (SHaRe registry member), University Hospital of Niguarda, Policlinico di Bari.

2) International Centers, all part of "SHaRe AC-DCM registry": Brigham and Women's Hospital, Stanford University Medical Center, University of Michigan, University of Pennsylvania Medical Center, Royal Brompton Hospital, Northwestern University, Maastricht University, University of Colorado

List of minor contributors from Italian TTN Consortium and DCM/AC SHaRe: Francesca Girolami, MD, Michele Lo Casto, MD, Antonella Mancinelli MD, Samuela Carigi MD, Irene Ruotolo, MD, Chiara Collesi PhD, Nicoletta Botto, PhD.

Burden Testing and mutation rate analyses

Raw genetic sequencing data and the cumulative number of patients referred for genetic testing, with DCM/NDLVC phenotypes, were entirely available from two of the centers (Trieste n=1205, Maastricht n=795). Median values of age at diagnosis, left ventricular ejection fraction and end diastolic diameter (LVEDD), as well as the percentage of ICD recipients were also available.

These cases (n=2000) were used to create a study left-ventricular cardiomyopathy case cohort.

The burden of rare (GnomAD Filtering Allele Frequency (FAF)<0.000084) TTNtv in exons with PSI>90 was calculated and compared with a Control cohort composed by individuals from the population of GnomAD v2.1 exomes (n=798504). The comparison was made separately for different *TTN* bands and their longer exons (Exon 48 for I band, 326 for A band and 386 for M band), as previously described.

The frequencies of TTNtv [(n° of rare TTNtv/coding length in bp)/n° population] and of probands [(n° of probands/coding length in Mbp)/n° population] was also separately calculated in different bands and exons both in cases and controls and compared.[8]

Collected data

Demographic, clinical, imaging, familial and therapeutic information was collected at baseline and at the last available evaluation.
At enrollment, clinical data included symptoms (dyspnea, syncope, palpitations, chest pain), presentation with ventricular arrhythmias (SCD, resuscitated cardiac arrest) or any other onset manifestations (for example, thromboembolism, accidental imaging finding).
The presence of cardiovascular risk factors (diabetes, Hypertension, coronary artery disease), competitive sport activity levels, and other triggers potentially facilitating TTN-induced DCM (such as alcohol excess, i.e., >80 g/day over a period of at least 5 years, chemotherapy, or pregnancy) was also documented.
Family members were recruited only if affected (presenting clinical or subclinical signs of a CMP: wall motion abnormalities, global dysfunction, myocardial fibrosis, early onset arrythmias).[2]
Age of onset was defined based on clinical diagnosis of CMP.
Data from 12-lead ECGs, and Holter ECG monitoring were recorded. Holter ECG data included premature ventricular contractions (PVCs, considered as a categorical variable as < or > 1,000/24hrs), non-sustained ventricular tachycardias (categorical) and atrial fibrillation (>30 sec of duration).
Echocardiographic biventricular dimensions and systolic function were assessed at transthoracic echocardiography as currently recommended by international guidelines. [17]
For the subgroup of patients with available cardiac magnetic resonance (CMR) parameters, the presence of late gadolinium enhancement (LGE), edema, and/or fat was recorded. If LGE was present, then distribution (septal, inferoposterolateral, and/or anteroapical), and pattern (midwall, subepicardial, or both) were further specified.
Based on their presenting phenotypes, after exclusion of secondary causes of cardiac disease, patients with TTNtv were classified into mutually exclusive phenotypic categories based on current international consensus guidance: Dilated Cardiomyopathy (DCM), Non-Dilated Left Ventricular Cardiomyopathy (NDLVC), Hypertrophic Cardiomyopathy (HCM), Arrhythmogenic Right Ventricular Cardiomyopathy (ARVC), Restrictive Cardiomyopathy (RCM) and congenital cardiomyopathy. [2]
Patients were considered to have left ventricular reverse remodeling (LVRR) if left ventricular ejection fraction normalized (LVEF ≥50%) or increased by ten absolute points (e.g. from LVEF 30% to LVEF 40%) in a timespan between three and 24 months of follow-up. [18]

Study outcomes

MVA comprise ventricular fibrillation, sustained ventricular tachycardia (i.e. lasting > 30 seconds or with hemodynamic instability), appropriate ICD interventions (either shock or anti-tachycardia pacing on ventricular fibrillation or sustained VT), and aborted sudden cardiac death (SCD). SCD was defined as witnessed SCD, with or without documented ventricular fibrillation, within one hour of acute symptoms, or nocturnal death with no antecedent history of immediate worsening symptoms.

Statistical Analysis

Variables were expressed as mean ± standard deviation or median and inter-quartile range for continuous variables, or counts and percentage for categorical variables, as appropriate.

Continuous variables were compared using either the one-way ANOVA test or the non-parametric Mann–Whitney U test, while chi-square or Fisher exact tests were used to compare discrete variables. Bonferroni correction was applied when comparing the three band groups.

Burden comparisons to test for enrichment were performed using one-sided Fisher's exact test. Kaplan-Meier curves were estimated for the primary composite outcome, while cumulative incidence functions (CIF) were estimated for the two secondary outcomes. Kaplan-Meier curves and CIF were estimated for TTNtv in Z-I, A and M band and compared by the log rank test.

For the SCD/MVA outcome, cumulative incidence curves were obtained, accounting for death from other causes and HF/HT as competing events and compared using Gray's test. The same approach was applied to the HF/HT endpoint, where death from other causes and SCD/MVA were considered competing events. Cause-specific Cox proportional hazards mixed-effects models were also obtained to include other risk factors. To account for familial relationships, family membership was treated as a random effect. Patient origin, categorized as Italian or non-Italian, was addressed using a binary stratification term.

A $p\text{-value}<0.05$ was considered statistically significant. Statistical analyses were performed in R version 3.6.2 (R Foundation for Statistical Computing, Vienna, Austria).

2. SUPPLEMENTAL TABLES

Supplemental Table 1. List of TTN truncating variants of the study. Genomic coordinates are relative to the GRCh38 reference genome assembly, and transcript changes refer to transcript NM_001267550.2 (ENST00000589042). Exon number notation refers to the meta transcript (canonical), as sourced by TTN db (https://www.cardiodb.org/titin/titin_transcripts). In the case of essential splice site variants at intronic nucleotides, the exon at the involved splice junction is reported. All reported variants are observed in heterozygosity.

**c.**	**p.**	**exon**	**Band**	**N° cases**
c.1638del	p.Lys546Asnfs*5	10	Z	2
c.1758_1763delAACTACinsT	p.Glu586Aspfs*12	11	Z	1
c.4027C>T	p.Gln1343*	24	Z	1
c.5658delG	p.Arg1886Serfs*25	28	Z-I	1
c.6248del	p.Arg2083Lysfs*56	28	Z-I	2
c.6848C>A	p.Ser2283*	30	I	1
c.7148delG	p.Ser2383Metfs*7	31	I	4
c.7855+1G>T	p.?	33	I	1
c.8220G>A	p.Trp2740*	35	I	1
c.10165+2T>C	p.?	44	I	1
c.10241_10247del	p.Tyr3414Leufs*7	44	I	1
c.11919del	p.Glu3974Lysfs*62	48	I	3
c.12195del	p.Asp4066Thrfs*10	48	I	1
c.12316C>T	p.Gln4106*	48	I	1
c.12405del	p.Asn4135Lysfs*33	48	I	1
c.12478del	p.Thr4160Profs*8	48	I	2
c.12587C>A	p.Ser4196*	48	I	1
c.12873del	p.Pro4292Leufs*13	48	I	1
c.12943_12950delGGGCAAGA	p.Gly4315Phefs*Ter6	48	I	1
c.13100del	p.Lys4367Argfs*27	48	I	11

c.13335del	p.Thr4446Leufs*6	48	I	1
c.13696C>T	p.Gln4566*	48	I	1
c.13943_13947dup	p.Phe4650Lys*14	48	I	1
c.14237del	p.Val4746Alafs*23	49	I	3
c.14371+1G>A	p.?	49	I	1
c.41609-2A>C	p.?	227	I	1
c.41641C>T	p.Arg13881*	227	I	2
c.41974_41977del	p.Asp13992Phefs*7	228	I	1
c.42024+2T>C	p.?	228	I	1
c.42151G>T	p.Glu14051*	229	I	1
c.42263_42266dup	p.Asn14089Lysfs*14	230	I	2
c.42267del	p.Asp14090Ilefs*2	230	I	1
c.42445_42446insTTT	p.Leu14148_Glu14149insVal*	231	I	1
c.42508dup	p.Met14170Asnfs*9	231	I	2
c.42512_42513insCTCCACACCCCCAGGAGAGACTCTAGAGCGATA	p.His14171_Val14172insSerThrProProGlyGluThrLeuGluArgTyr	231	I	1
c.44272C>T	p.Arg14758*	239	I	1
c.44639dup	p.Asn14880LysfsTer3	242	I	1
c.44958del	p.Lys14986Asnfs*30	244	I	1
c.45108del	p.Ala15038Profs*11	245	I	1
c.45189G>A	p.Trp15063*	245	I	1
c.38221A>T	p.Lys15309*	248	I	1
c.46179dup	p.Val15394Cysfs*11	248	I	1
c.46336_46339 dup	p.Leu15447Gln fs*11	249	I	1
c.46696+2T>C	p.?	250	I	3
c.47137_47138del	p.Ser15713Leufs*2	252	A	4
c.47494C>T	p.Arg15832*	253	A	2
c.48250G>T	p.Gly16084ter	257	A	1
c.48407del	p.Gly16136Alafs*12	258	A	1

c.48627_48630dup	p.Gly16211Asnfs*8	259	A	1
c.49171C>T	p.Arg16391*	262	A	2
c.49286dup	p.Asn16429Lysfs*7	262	A	1
c.49511del	p.Pro16504Glnfs*2	263	A	2
c.49648+2del	p.?	264	A	5
c.49813del	p.Val16605Phefs*24	265	A	1
c.49905dup	p.Pro16636Thrfs*9	265	A	2
c.49815del	p.Thr16607Profs*22	265	A	1
c.50134_50137dup	p.Thr16713Serfs*6	266	A	1
c.50170C>T	p.Arg16724*	266	A	1
c.50247_50248+2del	p.?	266	A	1
c.50249-1G>A	p.?	267	A	1
c.50296C>T	p.Arg16766*	267	A	1
c.50355-1G>C	p.?	268	A	2
c.51436+1G>A	p.?	271	A	1
c.51436C>T	p.Gln17146*	271	A	1
c.51624_51654dup	p.Gln17219Serfs*5	272	A	1
c.51667C>T	p.Arg17223*	272	A	1
c.51739+1G>C	p.?	272	A	1
c.51958C>T	p.Gln17320*	273	A	2
c.52171G>T	p.Glu17391*	274	A	1
c.52334_52335del	p.Val17445Glufs*3	274	A	1
c.52771del	p.Glu17591Asnfs*33	276	A	1
c.53003-1G>A	p.?	277	A	1
c.53182G>T	p.Glu17728*	277	A	3
c.53227_53232del	p.Val17743Hisfs*4	277	A	1
c.53259del	p.Lys17753Asnfs*7	277	A	1
c.53653G>T	p.Glu17885*	279	A	1
c.53918del	p.Gly17973Glufs*18	280	A	2

c.54000G>A	p.Trp18000*	280	A	2
c.54067C>T	p.Arg18023*	280	A	1
c.54112del	p.Glu18038Argfs*47	280	A	2
c.54418C>T	p.Arg18140*	282	A	3
c.54602_54608dup	p.Glu18204Leufs*16	282	A	1
c.54638G>A	p.Trp18213*	282	A	1
c.54652C>T	p.Arg18218*	282	A	1
c.54768del	p.Ser18258Valfs*34	282	A	1
c.54811+1G>A	p.?	282	A	1
c.81755G>A	p.Trp18187*	282	A	1
c.55269+3A>C	p.?	284	A	1
c.55332del	p.Ile18445Leufs*42	286	A	5
c.55869dup	p.Thr18624Tyrfs*13	288	A	1
c.55942dup	p.Asp18648Glyfs*9	288	A	2
c.56732dup	p.Asp18911Glufs*25	291	A	1
c.56806C>T	p.Arg18936*	291	A	2
c.57331C>T	p.Arg19111*	294	A	5
c.57847+5_57847+8del	p.?	295	A	1
c.58066_58067insC	p.Glu19356Alafs*27	296	A	1
c.58270G>T	p.Glu19424*	297	A	1
c.58327del	p.Ala19443Leufs*2	297	A	4
c.59201_59202del	p.Pro19734Argfs*5	300	A	1
c.59492_59495del	p.Ala19831Glufs*4	301	A	1
c.59570_59571insAlu	p.Leu19857Phefs*45	301	A	1
c.59780_59781insTG	p.Leu19927Profs16aaX	302	A	2
c.59866C>T	p.Gln19956*	302	A	2
c.59926+1G>A	p.?	302	A	1
c.60865_60866insA	p.Thr20289AsnfsTer10	304	A	1
c.60931C>T	p.Arg20311*	304	A	3

c.61437_61441dup	p.Thr20481Metfs*10	304	A	1
c.61682C>G	p.Ser20561*	304	A	1
c.61876C>T	p.Arg20626*	304	A	6
c.61921C>T	p.Arg20641*	304	A	2
c.62217T>A	p.Tyr20739*	304	A	1
c.62246del	p.Gly20749Valfs*7	304	A	1
c.62337_62340del	p.Thr20780Serfs*32	304	A	1
c.62830_62834del	p.K20944Rfs*9	304	A	1
c.62909dup	p.Glu20971Argfs*4	304	A	1
c.63601C>T	p.Arg21201*	306	A	2
c.63625C>T	p.Arg21209*	306	A	1
c.63794-1G>A	p.?	307	A	11
c.64423C>T	p.Gln21475*	309	A	2
c.64453C>T	p.Arg21485*	309	A	1
c.64577del	p.Ile21526Lysfs*4	309	A	3
c.64688del	p.Pro21563Leufs*10	310	A	1
c.64999C>T	p.Arg21667*	311	A	1
c.65858_65861dupTGTT	p.Leu21954Phefs*4	313	A	3
c.66020_66021del	p.Ser22007Cysfs*18	314	A	4
c.66378T>A	p.Tyr22126*	315	A	1
c.66498T>G	p.Tyr22166*	316	A	2
c.66628C>T	p.Gln22210*	316	A	1
c.66859G>T	p.Gly22287*	317	A	1
c.67028dup	p.Glu22344Glyfs*10	317	A	1
c.67058-1G>C	p.?	318	A	1
c.67276delinsTAA	p.Val22426ter	318	A	2
c.67348+1G>A	p.?	318	A	1
c.67480_67484del	p.Glu22494*	319	A	1
c.67495C>T	p.Arg22499*	319	A	3

c.67667del	p.Pro22556Leufs*19	320	A	1
c.67802C>A	p.Ser22601*	320	A	1
c.67863C>G	p.Tyr22621*	320	A	2
c.68124_68125del	p.His22709*	320	A	1
c.68195C>A	p.Ser22732*	320	A	1
c.68243del	p.Pro22748Hisfs*15	321	A	1
c.68276C>G	p.Ser22759*	321	A	1
c.68388_68391dup	p.Ile22798Serfs*8	322	A	3
c.68508dup	p.Val22837Cysfs*7	322	A	1
c.61193T>A	p.Leu22966*	324	A	1
c.69065_69084dup20	p.Lys23029Leufs*26	324	A	2
c.69086dup	p.Val23030Glyfs*10	324	A	1
c.69241_69242del	p.Glu23081Asnfs*11	324	A	1
c.69379C>T	p.Gln23127*	324	A	1
c.69412+1G>C	p.?	324	A	1
c.69458_69461dup	p.Asn23154Lysfs*14	325	A	1
c.69557G>A	P.Trp23186*	325	A	1
c.69783G>A	p.Trp23261*	326	A	1
c.69923_69926dup	p.Lys23309Asnfs*2	326	A	1
c.70083_70086dup	p.Arg23363Tyrfs*2	326	A	2
c.70215dup	p.Asn23406*	326	A	1
c.70885del	p.Met23629Cysfs*37	326	A	1
c.70940del	p.Ala23647Valfs*19	326	A	2
c.70978C>T	p.Arg23660*	326	A	2
c.71602C>T	p.Arg23868*	326	A	1
c.71668G>T	p.Gly23890*	326	A	2
c.71690_71691dup	p.Glu23898Metfs*5	326	A	1
c.71832dup	p.Trp23945Metfs*3	326	A	1
c.71980_71986delinsTA	p.Ala23994*	326	A	1

c.72226_72229dup	p.Glu24077Valfs*11	326	A	1
c.72263del	p.Val24088Glufs*45	326	A	1
c.72542dup	p.Leu24182Alafs*5	326	A	1
c.72669del	p.Asp24224Ilefs*8	326	A	1
c.72707_72710dup	p.Asp24237Glufs*12	326	A	1
c.72713C>A	p.Ser24238*	326	A	1
c.72777_72783del	p.Glu24260Valfs*19	326	A	1
c.72956_72963del	p.Asp24319Valfs*2	326	A	2
c.73075_73076dup	p.Met24359Ilefs*45	326	A	1
c.73332C>A	p.Cys24444*	326	A	1
c.73570del	p.Gln24524Argfs*3	326	A	3
c.73816_73819del	p.Pro24606Leufs*16	326	A	1
c.73846C>T	p.Arg24616*	326	A	1
c.74368_74376delinsTAAG	p.Leu24790*	326	A	1
c.76654C>T	p.Arg25552*	326	A	4
c.76961dup	p.Asn25654Lysfs*4	326	A	1
c.77098_77099dup	p.Gln25700Hisfs*7	326	A	2
c.77299dup	p.Tyr25767Leufs*4	326	A	3
c.77334del	p.Arg25779Glufs*11	326	A	3
c.77345_77346insATAT	p.Arg25783Tyrfs*16	326	A	1
c.77518C>T	p.Gln25840*	326	A	1
c.78342T>G	p.Tyr26114*	326	A	1
c.78381dup	p.Arg26128Thrfs*2	326	A	1
c.78923G>A	p.Trp26308*	326	A	1
c.79273A>T	p.Lys26425*	326	A	1
c.79639C>T	p.Arg26547*	326	A	2
c.79678A>T	p.Lys26560*	326	A	1
c.79684C>T	p.Arg26562*	326	A	1
c.79801G>T	p.Gly26601*	326	A	1

c.80494G>T	p.Glu26832*	326	A	1
c.80899_80900del	p.Val26967Tyrfs*16	326	A	4
c.81650G>A	p.Trp27217*	326	A	1
c.81943G>T	p.Glu27315*	326	A	1
c.81988C>T	p.Gln27330*	326	A	1
c.82313dup	p.Asn27438Lysfs*2	326	A	1
c.82350T>G	p.Tyr27450*	326	A	1
c.82525C>T	p.Arg27509*	326	A	1
c.82536del	p.Gly27513Alafs*12	326	A	1
c.82657G>T	p.Gly27553*	326	A	1
c.82883del	p.Lys27628Argfs*94	326	A	1
c.83048del	p.Lys27683Argfs*40	326	A	1
c.83104C>T	p.Arg27702*	326	A	1
c.83393_83396del	p.Arg27798Ilefs*24	326	A	3
c.83576_83577del	p.Lys27859Serfs*3	326	A	1
c.83860del	p.Val27954*	326	A	1
c.84286G>T	p.Glu28096ter	326	A	3
c.84481_84488del	p.Pro28161Cysfs*17	326	A	1
c.84498_84514del	p.Thr28167Cysfs*8	326	A	2
c.84819G>A	p.Trp28273*	326	A	4
c.84992del	p.Asn28331Metfs*17	326	A	1
c.85011_85014del	p.Glu28338Hisfs*9	326	A	1
c.85090C>T	p.Arg28364*	326	A	1
c.85207A>T	p.Arg28403*	326	A	4
c.85612_85619del	p.Glu26897Serfs*37	326	A	1
c.85841dup	p.Leu28615Thrfs*6	326	A	1
c.85969A>T	p.Lys28657X	326	A	2
c.86052dup	p.Gly28685Trpfs*10	326	A	1
c.86363G>A	p.Trp28788*	326	A	3

c.86465del	p.Lys28822Serfs*4	326	A	1
c.86640C>G	p.Tyr28880*	326	A	2
c.86675G>A	p.Trp28892*	326	A	1
c.86751del	p.Phe28917Leufs*21	326	A	1
c.86799_86802del	p.Gly28936*	326	A	1
c.86821+2T>A	p.?	326	A	1
c.86889del	p.Trp28963Cysfs*2	327	A	1
c.87040C>T	p.Arg29014*	327	A	1
c.87046_87049del	p.Phe29016Leufs*7	327	A	1
c.87451del	p.Thr29151Leufs*5	328	A	1
c.87624C>A	p.Tyr29208*	328	A	1
c.87707dup	p.Thr29237Asnfs*12	329	A	1
c.87782del	p.Pro29261Glnfs*10	329	A	5
c.88008_88009delTG	p.Cys29336*	329	A	1
c.88180C>T	p.Gln29394*	330	A	3
c.88344_88359del	p.Val29449Glnfs*5	331	A	5
c.88783del	p.Thr29595Profs*17	332	A	1
c.88956del	p.Val29653Tyrfs*13	333	A	1
c.88996del	p.Ser29666Valfs*13	333	A	2
c.89197_89197+2del	p.?	333	A	1
c.89870dup	p.Leu29958Serfs*3	335	A	1
c.90495del	p.Trp30165Cysfs*2	335	A	1
c.90830_90831insC	p.30277fs*10aaX	335	A	1
c.90924_90925insT	p.Sln30309Serfs*31	335	A	1
c.91476T>G	p.Tyr30492*	336	A	2
c.91572_91575del	p.Ile30525*	337	A	1
c.91615_91621del	p.Lys30539Glufs*18	337	A	1
c.92299_92300delinsTA	p.Val30767*	339	A	1
c.92299del	p.Val30767*	339	A	1

c.92317C>T	p.Arg30773*	339	A	1
c.92876G>A	p.Trp30959*	339	A	4
c.93166C>T	p.Arg31056*	339	A	2
c.93764_93765del	p.Arg31255Thrfs*2	339	A	1
c.93781C>T	p.Arg31261*	339	A	1
c.93897del	p.Phe31299Leufs*14	339	A	1
c.94055del	p.Gly31352Valfs*44	339	A	2
c.94344_94347del	p.Lys31448Asnfs*8	340	A	2
c.94816C>T	p.Arg31606*	341	A	2
c.94855C>T	p.Arg31619*	342	A	1
c.95008C>T	p.Arg31670*	342	A	1
c.95164C>T	p.Gln31722*	343	A	1
c.95341C>T	p.Arg31781*	343	A	1
c.95469dup	p.Ile31824Tyrfs*9	344	A	1
c.95638C>T	p.Gln31880*	344	A	2
c.95735del	p.Pro31912Hisfs*31	345	A	1
c.95805C>A	p.Tyr31935*	345	A	1
c.95966del	p.Asn31989Thrfs*2	345	A	1
c.96143G>A	p.Trp32048*	346	A	1
c.96260_96279dup	p.Lys32094Leufs*26	346	A	4
c.96407del	p.Pro32136Glnfs*14	347	A	1
c.96824_96831del	p.Phe32275*	347	A	1
c.97050dup	p.Glu32351Argfs*6	348	A	1
c.97051G>T	p.Glu32351*	348	A	1
c.97427dup	p.Thr32477Asnfs*13	349	A	5
c.97555_97558del	p.Thr32519Glyfs*12	350	A	1
c.97595del	p.Gly32532fs	350	A	1
c.97629del	p.Asp32544Ile*6	350	A	1
c.97967_97974del	p.Lys32656Argfs*21	351	A	2

c.98299_98300del	p.Arg32767Glyfs*2	352	A	4
c.99034A>T	p.Lys33012*	354	A	1
c.99181_99182dupGG	Gly33062Glu fs*21	354	A	1
c.99256A>T	p.Arg33086*	354	A	1
c.99785_99796delinsTTCTT	p.Lys33262Ilefs*36	355	A-M	1
c.100004del	p.Glu33335Glyfs*14	356	A-M	1
c.100217_100218del	p.Lys33406Argfs*13	357	A-M	1
c.100825C>T	p.Arg33609*	358	M	1
c.101098delinsCT	p.Asp33700Leufs*13	358	M	2
c.102097_102101dup	p.Phe34034Leufs*23	358	M	2
c.102510G>A	p.Trp34170*	358	M	1
c.102999del	p.Val34334Trpfs*15	358	M	3
c.103360del	p.Glu34454Asnfs*3	358	M	1
c.103360delG	p.(Glu34454Asnfs*3	358	M	1
c.103599_103602del	p.Arg34534Asnfs*19	358	M	1
c.103792del	p.Ser34598Glnfs*8	358	M	1
c.104092delC	p.Arg34698Glufs*49	358	M	2
c.104540_104541insATAT	p.Tyr34847*	358	M	1
c.104800C>T	p.Gln34934*	358	M	1
c.106019del	p.Gly35340Valfs*65	358	M	1
c.106792C>T	p.Gln35598*	360	M	1
c.106954C>T	p.Arg35652*	360	M	1

Supplemental Table 2. Results of burden comparison for *TTN*tv between our case cohort of NDLVC/DCM (n=2,000) patients and gnomAD v4.1 (n=798,504).

**Band**	Exon	**Cases (probands) NDLVC/DCM case cohort (n= 2,000)**	prevalence NDLVC/DCM case cohort (%)	**Cases GnomAD v4.1 (n=798,504)**	prevalence GnomAD (%)	**OR (95% CI) NDLVC/DCM case cohort Vs GnomAD**	**EF (95% CI)**
**AM-M**		11	**0.55**	802	0.10	**5.5 (3.03-9.99)**	**0.82 (0.67 - 0.90)**
	**exon 358**	8	0.4	528	0.07	**6.7 (3.02-12.22)**	**0.84 (0.67 - 0.92)**
**A**		109	**5.45**	2593	0.32	**17.69 (14.53-21.5)**	**0.94 (0.93 - 0.95)**
	**exon 326**	31	1.55	612	0.08	**20.53 (14.27-29.52)**	**0.95 (0.93 - 0.97)**
**Z-I cons**		32	**1.6**	2510	0.31	**5.16 (3.63-7.33)**	**0.81 (0.72 - 0.86)**
	**exon 48**	12	0.6	166	0.02	**29.03 (16.13-52.24)**	**0.97 (0.94 - 0.98)**
**Total**		152	**7.6**	5,905	0.74	**11.04 (9.34-13.05)**	**0.91 (0.89 - 0.92)**

Clinical characteristics of NDLVC/DCM CMP sub-cohorts are herein listed. Maastricht (n=795) : Age (median, IQR): 55(46-63); LVEF: 32% (24-41); echo-indexed LVEDD, mm: 30 (25-34) (range: no/mildly dilated); ICD recipient: 37%; Trieste (n=1205): Age (median, IQR) 53 (44-65); echo-LVEF: 34% (25-45); echo-indexed LVEDD, mm: 32 (29-36) (range: mildly dilated); ICD recipient: 43 %. P-value for all comparisons <0.0001

Supplemental Table 3. Allele (tv) and case frequency (per Megabase) of *TTN* bands and exons, across different cohorts (DCM/NDLVC case cohort, GnomAD)

Band/Exon	coding lenght (bp)	n° TTNtv NDLVC/DCM case cohort (n= 2,000)	n° TTNtv in Gnomad v4.1 (n=798,504)	**allele frequency in NDLVC/DCM case cohort**	mutation rate GnomAD	**Case Frequency NDLVC/DCM case cohort (per Mb)**	**Case Frequency GnomAD (per Mb)**	
**AM-M**	8,111	10	285	6.16×10^{-7}	4.40×10^{-8}	0.68	0.12	
**exon_358**	5,609	7	174	6.23×10^{-7}	3.88×10^{-8}	0.71	0.12	
**A**	52,899	83	1,400	7.85×10^{-7}	3.14×10^{-8}	1.03	0.06	
**exon_326**	17,106	27	387	7.89×10^{-7}	2.83×10^{-8}	0.91	0.04	
**Z-I_cons**	19,909	24	670	6.02×10^{-7}	4.21×10^{-8}	0.80	0.16	
**exon_48**	2,781	5	96	9.98×10^{-7}	4.32×10^{-8}	2.16	0.07	
**total**	80,919	117	2,355	7.23×10^{-7}	3.64×10^{-8}	0.94	0.09	

Supplemental Table 4. Follow-up characteristics of the study population

Patients with complete follow up data	436 (93%)	Total	A band	I band	M band	p-value
**ICD implantation (primary prevention)**	436 (100%)	156 (36%)	122 (35%)	27 (40%)	7 (32%)	0.62
**ICD implantation (secondary prevention)**	436 (100%)	22 (5%)	13 (4%)	4 (6%)	5 (23%)	**0.003**
**Age at ICD implantation**	*104 (58%)*	55 [44-62]	54 [42-61]	61 [58-64]	57 [48-60]	**0.03**
**Age at ICD implantation (primary prevention)**	*83 (53%)*	55 [44-63]	54 [41-63]	61 [58-65]	59 [59-59]	0.08
**Age at ICD implantation (secondary prevention)**	*20 (91%)*	54 [45-58]	46 [43-57]	61 [58-62]	54 [46-61]	0.31
**LVEF at ICD implantation**	*81 (46%)*	33 [26-41]	31 [25-41]	34 [29-37]	61 [57-64]	**0.00**
**LVEF at ICD implantation (primary prevention)**	*66 (42%)*	31 [25-37]	31 [25-38]	34 [30-34]	0 [0-0]	0.77
**LVEF at ICD implantation (secondary prevention)**	*15 (68%)*	42 [35-50]	41 [35-45]	40 [32-44]	61 [57-64]	**0.03**

Supplemental Table 5. Break-down of SCD/MVA events

	All (n=436)	A-band	Z/I-band	M-band
**Total patients with SCD/MVA events**	**84**	**58**	**19**	**7**
**SCD events**	4 (5%)	4 (7%)	0 (0%)	0 (0%)
**aSCD events**	17 (20%)	10 (17%)	2 (11%)	5 (71%)
**Appropriate ICD interventions**	35 (42%)	24 (41%)	9 (47%)	2 (29%)
Shock:	22	17	4	1
ATP:	13	7	5	1
**Sustained VT events**	28 (33%)	20 (34%)	8 (42%)	0 (0%)

SCD: sudden cardiac death; MVA: major ventricular arrhythmias; aSCD: aborted sudden cardiac death; ICD: implantable cardioverter defibrillator; ATP: anti-tachycardia pacing; VT: ventricular tachycardia.

Supplemental Table 6. Multivariable MVA outcome analyses across different Bands

1) A Band: n= 278, number of events= 44
(69 observations deleted due to missing data)

**Characteristic**	**HR**	**95% CI**	**p-value**
Gender	3.95	1.37, 11.4	0.011
Age diagnosis	1.02	0.99, 1.04	0.2
LVEF at diagnosis	0.97	0.94, 1.0	0.018

HR = Hazard Ratio, CI = Confidence Interval, LVEF= left ventricular ejection fraction

2) Z/ I Band: n= 53, number of events= 13
(14 observations deleted due to missing data)

**Characteristic**	**HR**	**95% CI**	**p-value**
Gender	1.82	0.42, 7.87	0.4
Age diagnosis	1.02	0.96, 1.08	0.5
LVEF at diagnosis	0.94	0.87, 1.00	0.058

HR = Hazard Ratio, CI = Confidence Interval, LVEF= left ventricular ejection fraction

3) M band: n= 20, number of events= 6
(2 observations deleted due to missing data)

**Characteristic**	**HR**	**95% CI**	**p-value**
Gender	1.46	0.23, 9.37	0.7
Age diagnosis	0.96	0.88, 1.04	0.3
LVEF at diagnosis	1.06	0.97, 1.15	0.2

HR = Hazard Ratio, CI = Confidence Interval, LVEF= left ventricular ejection fraction

Supplemental Table 7. Brief description of patients experiencing SCD/MVA with a LVEF£35% prior to the event (time window of 6 months maximum), grouped by band variant. Cells are empty when information was not available.

**Band**	**Sex**	**LVEF at baseline**	**Last LVEF prior to SCD/MVA**	**Age at event (years)**	**NSVT at baseline**	**Left Ventricular Reverse Remodeling**
A	male	45	14	54		
A	male	17	17	21	no	no
A	male	25	19	74	yes	no
A	male	33	21	55	yes	no
A	male	29	23	56	no	no
A	female	15	23	76	no	yes
A	male	20	23	58	yes	yes
A	male	30	24	44	no	yes
A	male		25	58		no
A	male		25	65		no
A	male		25	55		no
A	female		25	54		no
A	female	25	25	57	yes	no
A	male	28	30	72	yes	no
A	male		30	60		no
A	male	33	30	64		yes
A	male	21	32	62	yes	no
A	male	20	35	55	yes	no
A	male		35	53	yes	no
A	male	15	35	42		yes
A	male	42	35	46	no	
A	male	34	35	56	yes	yes
I	male		15	67		no
I	male	20	20	62		no

I	male	15	32	42		no
I	male	20	33	64	yes	yes
I	male	25	33	61		yes
Z	female	29	32	51	yes	yes
Z-I	male	30	25	63	yes	yes
M	*none*					

3. SUPPLEMENTAL FIGURES

Supplemental Figure 1. Kaplan Mayer curves for primary outcome at 120 months stratified by TTNtv location.
Kaplan–Meier curves showing event-free survival for the composite primary outcome of all-cause death or heart transplantation over 120 months according to *TTN*tv location. No significant difference in survival between groups was observed (log-rank p = 0.44).

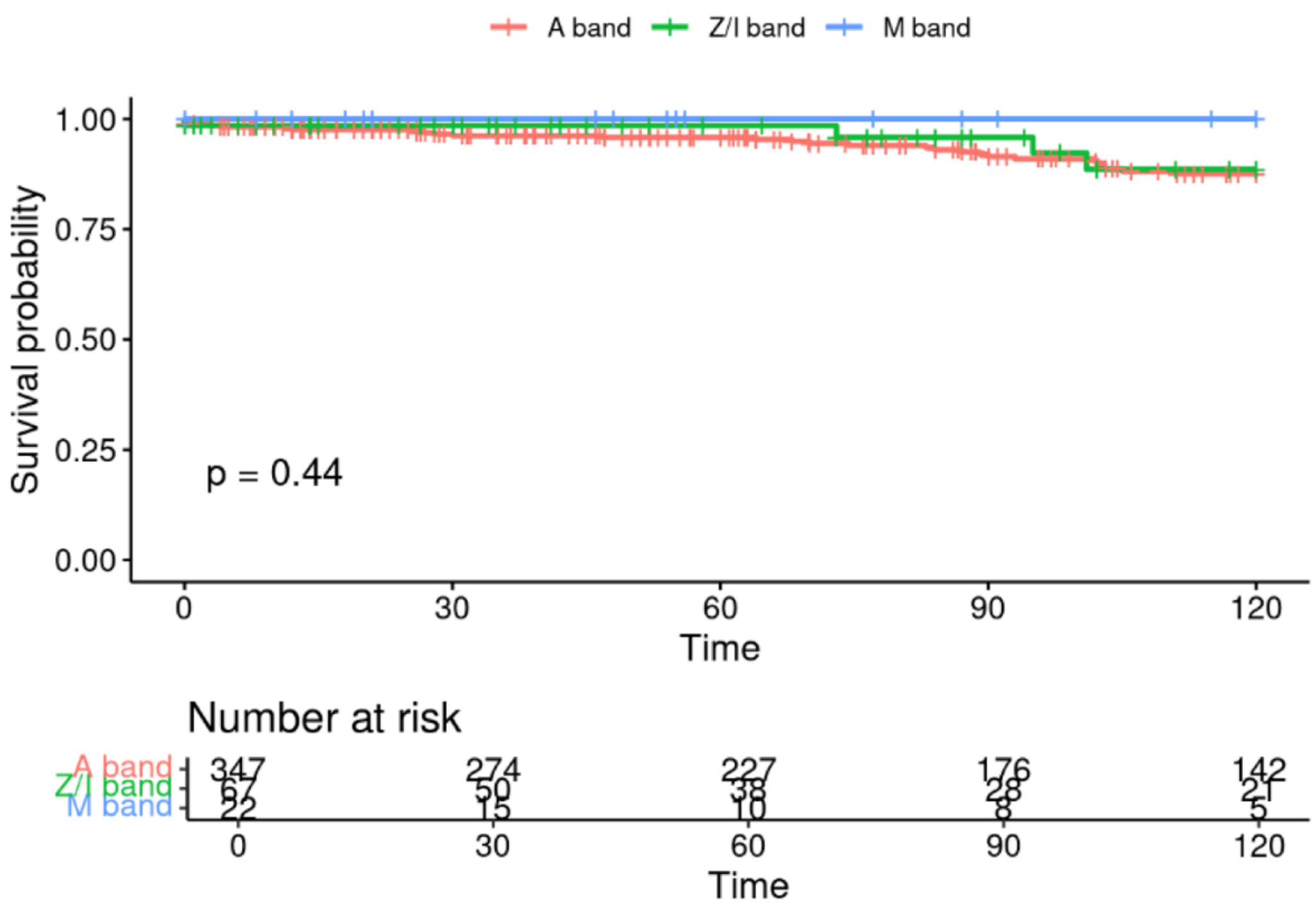

Supplemental Figure 2. CIF Curves for SCD/MVA in study population after excluding patients with MVA at disease onset.
A significant difference in event rates among groups was observed (Gray's test p = 0.031), with higher incidence in the M and Z/I- band groups compared with the A band.

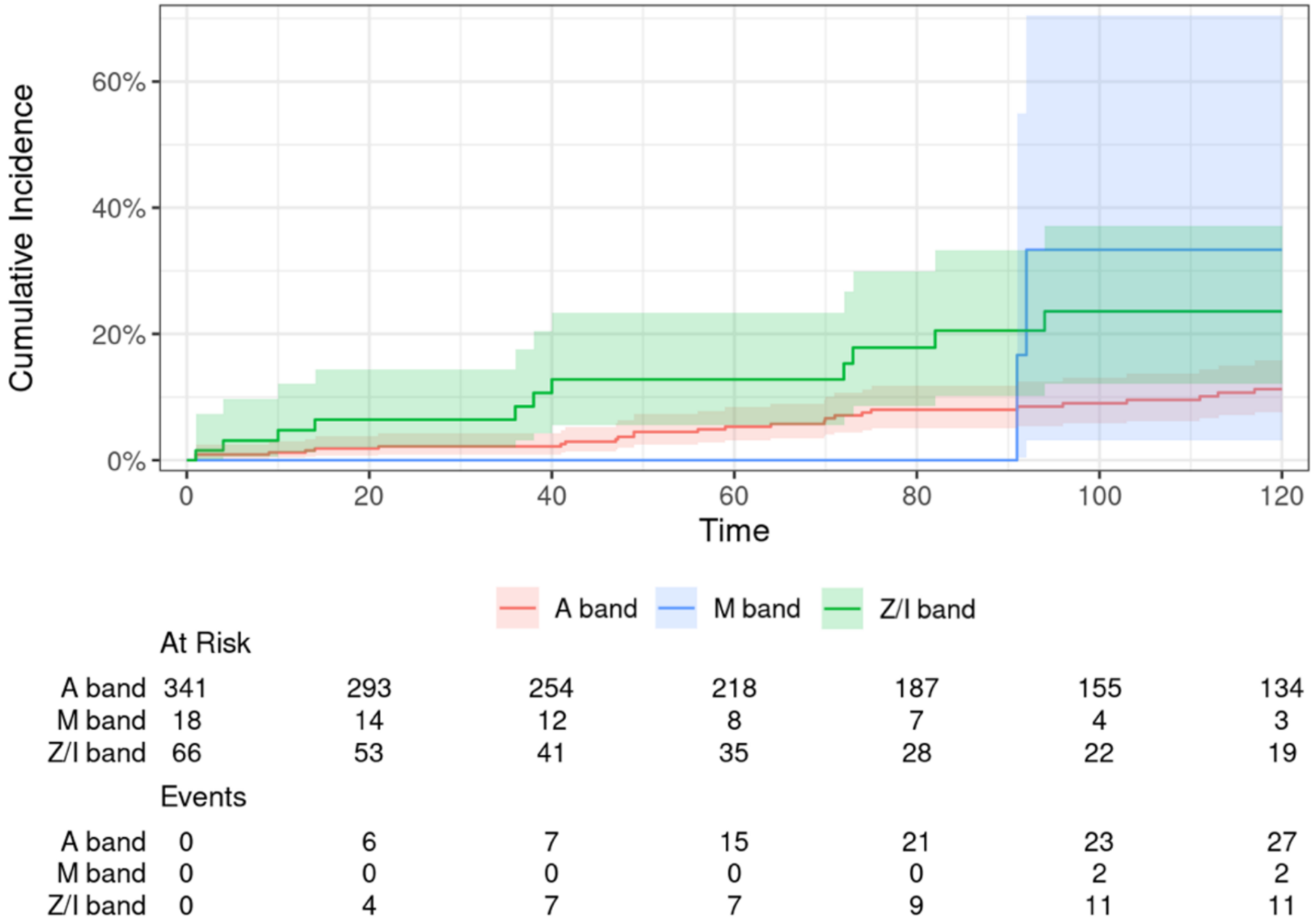

Characteristic	N	N Event	Month 120	p-value[1]
term	425	40		0.031
A band	341	27	11% (7.6%, 16%)	
M band	18	2	33% (3.2%, 70%)	
Z/I band	66	11	24% (12%, 37%)	
[1] Gray's Test				

Supplemental Figure 3. CIF Curves for SCD/MVA in probands only, after excluding subjects with MVA at disease onset. A significant difference in event rates among groups was observed (Gray's test p = 0.016), with higher incidence in the M and Z/I band groups compared with the A band.

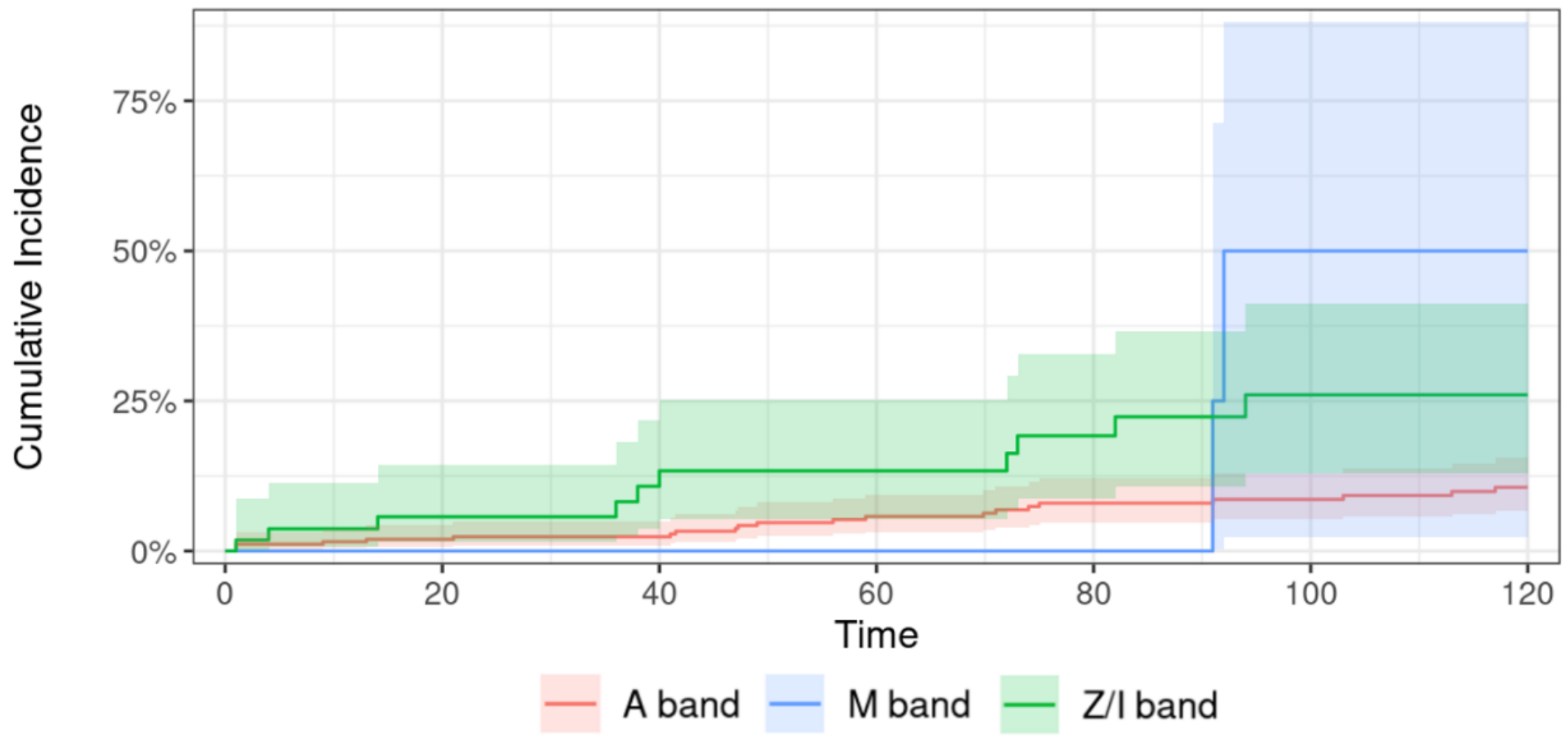

At Risk

	0	20	40	60	80	100	120
A band	264	231	204	177	153	129	113
M band	13	10	9	6	5	2	1
Z/I band	54	44	34	30	24	18	16

Events

	0	20	40	60	80	100	120
A band	0	5	6	13	17	18	21
M band	0	0	0	0	0	2	2
Z/I band	0	3	6	6	8	10	10

**Characteristic**	**N**	**N Event**	**Month 120**	**p-value**[1]
term	331	33		0.016
A band	264	21	11% (6.7%, 15%)	
M band	13	2	50% (2.3%, 88%)	
Z/I band	54	10	26% (13%, 41%)	

[1] Gray's Test

Supplemental Figure 4. CIF Curves for HF outcome in study population

No significant difference in HF incidence among groups was observed (Gray’s test p = 0.50); however, there was a trend toward higher event rates in the A and Z/I band groups compared with the M band.

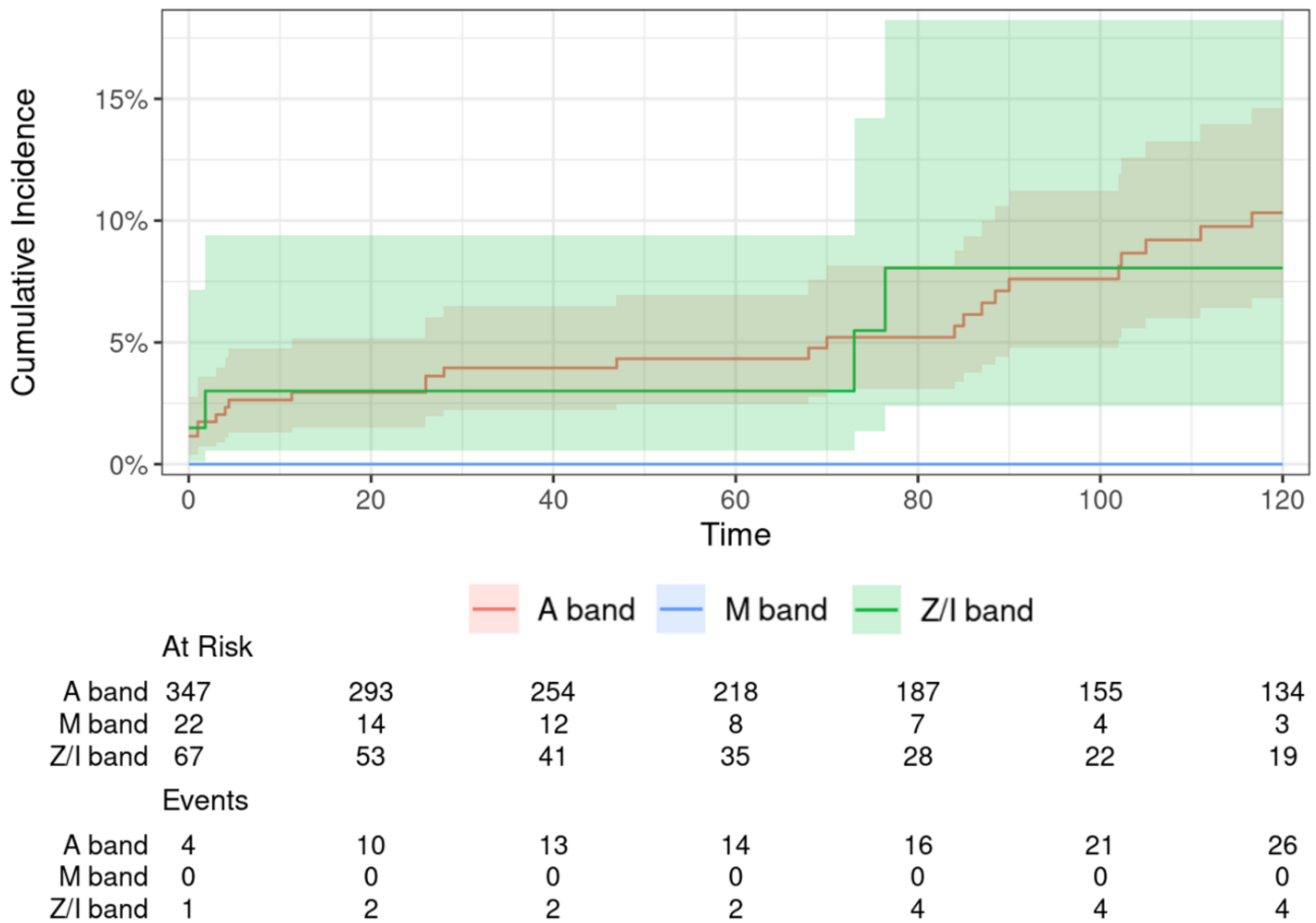

**Characteristic**	**N**	**N Event**	**Month 400**	**p-value**[1]
term	436	30		0.5
A band	347	26	10% (6.8%, 15%)	
M band	22	0	0.00% (—%, —%)	
Z/I band	67	4	8.1% (2.4%, 18%)	

[1] Gray’s Test

Supplemental Figure 5. Association between baseline LVEF and SCD/MVA risk at follow-up according *TTN*tv location

Restricted cubic spline analyses illustrating the relationship between baseline LVEF and arrhythmic risk during follow-up, stratified by *TTN*tv location. Lower baseline LVEF is associated with higher arrhythmic risk in the A and Z/I bands, whereas this association is not observed in the M band.

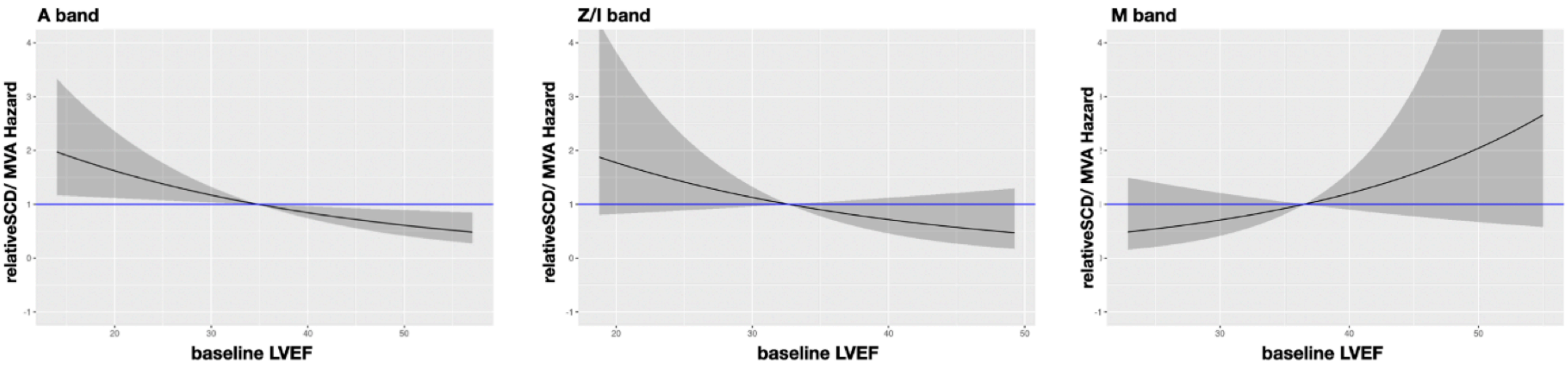

Supplement: Supplementary file 1 [file hcg-19-e005422-s001.pdf]
